# Development and validation of a vero cell-based suspension method for the detection of Zika virus

**DOI:** 10.17843/rpmesp.2023.403.12606

**Published:** 2023-09-25

**Authors:** Dina Popuche, Alfredo Huaman, Steev Loyola, María Silva, Sarah A. Jenkins, Carolina Guevara

**Affiliations:** 1 U.S. Naval Medical Research Unit SOUTH, Lima, Peru. U.S. Naval Medical Research Unit SOUTH Lima Peru; 2 Facultad de Medicina, Universidad Peruana Cayetano Heredia, Lima, Peru. Universidad Peruana Cayetano Heredia Facultad de Medicina Universidad Peruana Cayetano Heredia Lima Peru; 3 Vysnova Partners Inc., Maryland, USA Vysnova Partners Inc. Maryland USA; 4 Naval Medical Research Center, Silver Spring, USA Naval Medical Center Naval Medical Research Center Silver Spring USA

**Keywords:** Zika Virus, Zika Virus Infection, Virus Replication, Cell Culture Techniques

## Abstract

**Objective.:**

To develop and validate a cell suspension method using Vero 76 cells for culturing Zika virus (ZIKV) based on infection of detached freshly seeded cells.

**Material and methods.:**

Three different multiplicities of infection of ZIKV were used to develop and compare this novel method to the standard confluent cell monolayer method. In addition, we preliminary validated the cell suspension method using well-characterized ZIKV positive and negative clinical samples. The standard confluent cell monolayer method was used as the reference method, and viral isolation was confirmed by a ZIKV-specific RT-PCR. The sensitivity and its 95% confidence intervals for the cell suspension method were estimated. Also, a technical comparison of the cell suspension method against the cell monolayer method was performed.

**Results.:**

Our findings suggested that both the viral load and replication of ZIKV were comparable between both monolayer- and suspension-infection methods. Although both methods were suitable for culturing and isolating ZIKV, the cell suspension method was easier, cheaper, and quicker as well as a sensitive isolation technique. The cell suspension method was significantly more sensitive in detecting Zika in inconclusive cases by RT-PCR, with a fourfold increase compared to the confluent cell monolayer method.

**Conclusion.:**

The cell suspension method has the potential to be an effective method for cultivating and isolating ZIKV and its application is potentially useful in both research and clinical settings.

## INTRODUCTION

Zika virus (ZIKV) is an emerging flavivirus transmitted by Aedes mosquitoes in several Latin American countries ^1^^,^^2^. Since 2015, several countries reported autochthonous transmission of ZIKV that led to severe outbreaks in the Americas ^3^^-^^5^. ZIKV infection causes clinical manifestations in about 20 percent of patients who presented an acute onset of fever, maculopapular rash/eruptions, arthralgia, or conjunctivitis ^3^^,^^6^. After the emergence of ZIKV in Brazil in 2015, infection by the virus was associated with a significant increase in the number of infants born with microcephaly and birth defects, as well as an increase in neurological disease cases in all age groups, such as myelitis and Guillain-Barre ^7^^,^^8^. Several studies have shown that ZIKV can be transmitted through sex and blood transfusion, which complicates the understanding of the transmission process as infected individuals may not be aware of the transmission potential ^9^^-^^13^. Since ZIKV was declared as a global emergency by the World Health Organization, countries vulnerable to ZIKV emergence should be able to detect and confirm ZIKV cases regardless of whether or not active cases are detected in endemic regions ^14^^-^^16^.

Diagnostic testing for arboviral infections is aimed at the detection of the virus or host immune antibody response ^17^. Serological assays have been used to detect ZIKV IgM antibodies to confirm a recent infection, but the results of these tests are difficult to confirm due to cross-reactivity among flaviviruses and false positives ^18^^,^^19^. ZIKV is usually detected by real-time reverse transcription-polymerase chain reaction (RT-PCR) in serum, blood, urine, and other body fluids, but the use of molecular diagnostic tools may be limited as the kinetics of viremia and specific viral RNA sequences may not be specific enough to detect the virus ^20^. As such, negative or inconclusive results based on molecular assays do not necessarily imply the absence of ZIKV infection ^16^^,^^18^^,^^21^^,^^22^.

The different types of cell cultures, used in several virus isolation techniques, play an important role in diagnosing cases when samples of clinically suspected cases are negative or inconclusive by RT-PCR. Virus isolation is particularly sensitive and useful for detecting circulating viruses during the viremic stage of infection and for correctly classifying samples with inconclusive results but has drawbacks in settings with limited resources. Although virus isolation can be cost-effective and can be used to evaluate the kinetics of viral infections and clinical outcomes, it requires highly skilled operators and can be laborious ^15^^,^^17^. Viral isolation of ZIKV has been comparatively difficult ^20^^,^^23^ because the method relies on infrastructure and specific laboratory resources that are not available in developing countries, such as cold-chain processes for viral preservation, cell-culture laboratories and supplies, cell lines, and well-trained and competent laboratory personnel. Thus, diagnostic methods must be accessible and balanced. Having confident methods of ZIKV diagnosis is a time-sensitive need because susceptible populations like pregnant women and infants are at risk of ZIKV infection ^7^^,^^11^^,^^16^^,^^24^.

This study aimed to find an isolation method that balances the critical need for accuracy and the rapid detection of ZIKV, particularly during emergency or outbreak situations in which early detection and isolation are required for the development or evaluation of different diagnostic tests. For instance, complementary methods are required to rapidly detect or isolate the virus in cases when ZIKV infection is suspected with negative or inconclusive RT-PCR results. In this regard, conventional culture methods require a previously formed monolayer, which, depending on the concentration of seeded cells, could take between 12 or even 48 hours. As such, the development of isolation methods without the need for a pre-formed monolayer could have a significant impact on decreasing response times and could help decision-makers in developing better public health measures earlier. Here, we described the development and standardization of an in-house cell suspension method as well as its preliminary validation using human clinical samples. In addition, the cell suspension method was compared to the confluent cell monolayer method using Vero 76 cells to optimize an accurate and rapid alternative isolation technique for ZIKV detection.

**KEY MESSAGES**

Motivation for the study. To search and develop new useful alternatives for Zika virus (ZIKV) research in emergency situations.

Main findings. We developed and validated a novel method for isolating and culturing ZIKV based on infecting freshly seeded and unattached Vero cells. This method was found to be easy, cheap, fast and more sensitive when compared to the standard method used for ZIKV isolation.

Implications. The method we developed can be used to strengthen the diagnosis and public surveillance of ZIKV with fast and reliable results.

## MATERIAL AND METHODS

### Study design

Experimental study based on the design and validation of a two-phase proof-of-concept; *in vitro* experiment using a viral seed, and a preliminary validation with clinical samples. Both phases used Vero cells (African green monkey [*Chlorocebus*] renal epithelium) obtained from the American Type Culture Collection (ATCC; CRL-1587). We used 29 samples for preliminary validation, which were collected in Honduras (FHT codes, n=4), Colombia (FCC codes, n=4), Venezuela (FVM code, n=1) and Peru (FPI and FPY codes, n=20) by researchers from the U.S. Naval Medical Research Unit SUR. The samples were obtained from individuals with fever for up to five days and clinical symptoms compatible with Zika infection.

### Culturing cells for ZIKV inoculation

Vero 76 is an optimal cell line for ZIKV isolation and also supports the development of human viral vaccines and biomedical research on viral diseases ^18^^,^^25^^,^^26^. This cell line is used regularly in our laboratory for virus culturing and isolation because it is susceptible to flaviviruses, such as ZIKV and other arboviruses. Viability was assessed in all cell experiments using trypan blue in a hemocytometer, resulting in values between 90-95%. No differences in cell viability between cells used in the confluent cell monolayer or the cell suspension method were found.

*Confluent cell monolayer method:* Vero 76 cells were seeded at a density of 1.0 x 10^5^ cells/mL in a T12.5-culture flask (Corning, Glendale, AZ, United States; Cat. No.: 353018) using 3 mL of Eagle’s Minimum Essential Medium (EMEM) (Quality biological, Gaithersburg, MD, United States; Cat. No.: 112-018-101) supplemented with 10% fetal bovine serum (FBS) (Merck, Darmstadt, Germany; Cat. No.: F4135) and incubated for two days at 37°C, 5% CO_2_. Thereafter, the 2-day monolayer was confluent at 1.3 x 10^6^ cells per flask. This standard culturing method is used routinely to prepare cells for attempting to isolate ZIKV and other arboviruses.

*Cell suspension method:* On the day of inoculation of cells with ZIKV, Vero 76 cells were seeded in 12-well culture plates (Thermo Fisher, Cleveland, OH, United States; Cat. No.: 150628) using 1mL of EMEM supplemented with 10% FBS at a density of 2.0 x 10^5^ cells/mL. Prior to inoculation, cells were incubated for 30 minutes at 37°C, 5% CO_2_ to facilitate pH stabilization. This method was designed to eliminate the incubation time required to prepare a confluent cell monolayer in order to have a faster response time for ZIKV testing. However, it is important to mention that, as the incubation time elapses, the cells proliferate and tend to form a monolayer. The 12-well culture plates were selected because it allows for maximizing the use of cells and reagents per sample, and because it allows the inoculation of multiple samples per plate.

### Virus inoculation and follow-up

We used ZIKV isolated in Vero 76 cells collected from an acute serum sample obtained from a Zika case in Iquitos, Peru to carry out the viral infection for both methods. In order to assess viral replication, we prepared three multiplicities of infection (MOI) of 0.1, 0.01, and 0.001 in EMEM without FBS for virus inoculation of Vero 76 cells. Cell cultures without virus inoculum were used as negative controls.

*Monolayer-infection method:* After two days, the supernatant of Vero cells was discarded using a sterile technique. The cells were then inoculated with 0.2 mL of MOIs at concentrations of 0.1, 0.01, and 0.001 of ZIKV in duplicate. Flasks were shaken gently back and forth manually five times and incubated for 1 hour at 37°C in 5% CO_2_. Then, 3 mL of EMEM supplemented with 2% FBS was added to each culture and incubated under the same conditions described above. For this method, no media replacements were performed after the infection with the viral inoculum, thus, the incubation was continuous.

*Suspension-infection method:* Infection occurs in cells that were recently seeded and are not yet fully adhered to the culture plates. This method was standardized by inoculating 0.1 mL in duplicate of the three MOIs directly onto each Vero cell culture and then incubating for three days at 37°C in 5% CO_2_. A previous study suggested that, regardless of the viral titer used for the infection, the absence of FBS correlates with high rates of viral replication ^27^. Therefore, to avoid depletion of media components, on the third day, the medium was carefully removed using a 1-mL pipette without disturbing the monolayer and then replaced with 1 mL of new EMEM without FBS. Cultures were then placed in the incubator at 37°C in 5% CO_2_.

*Follow-up*: Vero cell cultures inoculated with ZIKV were observed daily for cytopathic effect (CPE) and 200 µL of supernatant of each inoculated culture were collected for further molecular and plaque assay analysis. Cultures were harvested when the cells showed >75% (3+) CPE or up to 10 days post-inoculation.

### Molecular assay

RNA was extracted using 140 µL from each supernatant sample obtained daily from ZIKV-infected Vero cells starting on day 0 up to the time of the appearance of CPE or up to 10 days post-inoculation. The QIAamp Viral RNA kit (Qiagen, Hilden, Germany, Cat. No.: 52904) was used for extraction according to the manufacturer’s instructions. Detection of ZIKV RNA was performed using primers and probes, and PCR conditions as previously described ^28^. The reaction mixture for the one-step RT-PCR was prepared using the Fast Virus 1-step master mix (Thermo Fisher, Cleveland, OH, United States; Cat. No.: 4444436), and the amplification was performed in the Applied Biosystems 7500 Fast Real-Time PCR Instrument (Thermo Fisher, Cleveland, OH, United States; Cat. No.: 4406984). The detection limit of the RT-PCR assay for ZIKV was previously described at the cycle threshold (Ct) of 36.2 ± 1.6 ^28^. Here, serum or blood samples with a Ct value less than or equal to the cutoff value were classified as positive (Ct ≤ 34.6), while those with no amplification signal were classified as negative. Samples with a late amplification signal (Ct > 34.6) were classified as inconclusive. The cycle threshold (Ct) values were registered and used as a reference for viral load.

### Plaque assay

**Figure 1:**
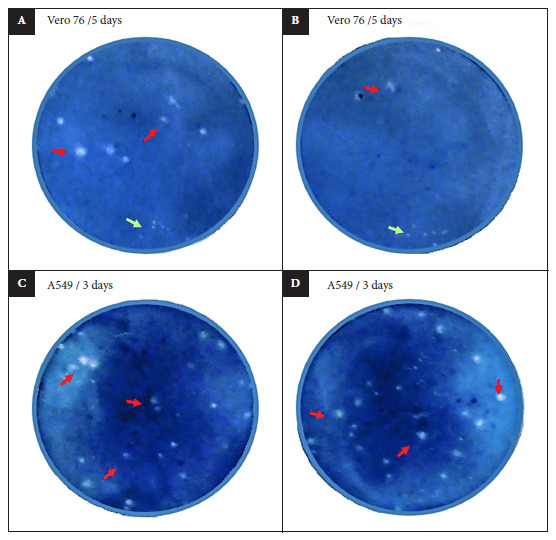
Zika virus (ZIKV) plaque assay in Vero 76 and A549 cells. The ZIKV seed was planted on Vero 76 and A549 cells to determine the optimal cell line for counting. All panels show cell monolayers stained on different days. The ZIKV plaques in Vero 76 (A and B) and A549 (C and D) cells were stained after 5 and 3 days post infection, respectively. Red arrows represent well-formed plaques considered for counting. Green arrows represent small and non-well-formed plaques.

A549 and Vero cells are susceptible to the ZIKV infection resulting in CPE ^25^. In previous experiments conducted in our laboratory, we did not find significant differences in the ZIKV load when comparing the plaque-forming unit counts in Vero and A549 cells using the semisolid plaque assay method ^29^. However, we noticed that plaques were better formed in A549 cells on the third day of infection in comparison to Vero cells (Figure 1). The semisolid plaque assay was carried out by preparing an A549 cell suspension in 12 well plates at a density of 2.0 x 10^5^ cells/mL per well. The cell cultures were placed in the incubator for 30 minutes at 37°C and 5% CO_2_. Cell supernatant samples were tested in a single 12-well culture plate, as in previous studies ^30^. Six 10-fold dilutions were prepared using 100 µL of each supernatant sample and then inoculated onto cultures in duplicates. After three hours of incubation under the same conditions described above, 1 mL of 3% of carboxymethyl cellulose was added to each culture and then incubated for three days. Cells were stained using 3 mL of a solution prepared with anhydrous sodium acetate, naphthol blue black, and acetic acid. Plaque forming units (PFU) were counted and used for calculating the PFU per mL (PFU/mL) to determine the infectivity titer.

### Validation of the cell suspension-infection method

Twenty-nine acute serum samples were used initially to validate the cell suspension method. This set of samples included 15 ZIKV-positive, 12 ZIKV-inconclusive, and 2 ZIKV-negative samples as determined by RT-PCR ^28^. ZIKV-inconclusive samples were included in the validation process under the assumption that the late amplification (39.0 > Ct > 34.6) observed in the RT-PCR was associated with a low viral load and that propagation in cell culture could subsequently help to classify the sample as positive or negative. The two negative samples were included as they showed a small amplification pattern close to 40 cycles. The blood specimens were collected from participants during the acute phase of febrile illness with clinical symptoms compatible with Zika infection from Honduras (FHT codes, n=4), Colombia (FCC codes, n=4), Venezuela (FVM code, n=1), and Peru (FPI and FPY codes, n=20). Collected samples were centrifuged to separate the serum and stored at -80°C until use. Thawed serum samples were diluted 1/10 using EMEM without FBS. The infection was carried out in the cell suspension infection and the confluent monolayer infection methods in duplicate as described above.

### Statistical analysis

PFU/mL values were transformed to log_10_ (PFU/mL). Ct and log_10_ (PFU/mL) values were plotted independently to assess the viral growth kinetics of the confluent cell monolayer and cell suspension method at the three MOI dilutions. We conducted Mann-Whitney tests using Ct and log_10_ (PFU/mL) values per day without considering the MOI inoculum to explore differences between methods. For the validation process, we explored differences in the harvest day using a paired T-test. The sensitivity and its 95% confidence intervals for both infection models were estimated considering the molecular assay as the gold standard. The data analysis was performed using Stata v16.0 (StataCorp. 2015. Stata Statistical Software: Release 14. College Station, TX: StataCorp LP.; licensed by Universidad Peruana Cayetano Heredia) and considering p<0.05 as significant.

### Ethical aspects

The study was conducted in accordance with the Declaration of Helsinki. The study protocols NMRCD.2010.0010 and NAMRU6.2020.004 were approved by the Institutional Review Board of the U.S. Naval Medical Research Unit SOUTH in compliance with all applicable federal and local regulations governing the protection of human subjects. During the first protocol, samples were collected from individuals who provided informed consent for arbovirus research, while for the second protocol, anonymized samples were used for the development and validation of laboratory-developed tests.

## RESULTS

### Technical analysis

**Figure 2:**
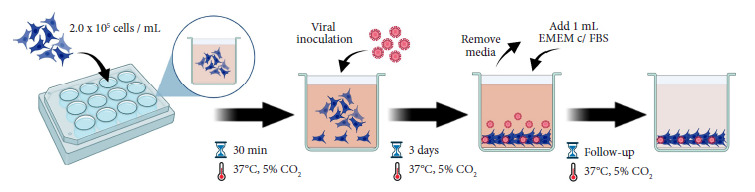
Overview of the culture and viral inoculation process of the cell suspension method. At the day of viral inoculation, Vero 76 cells were seeded in a 12-well culture plate at a density of 2.0 x 10^5^ cells/mL in Eagle’s Minimum Essential Medium (EMEM) supplemented with 10% fetal bovine serum (FBS). The plate was incubated for 30 minutes at 37°C, 5%CO^2^. A 1:10 dilution of each sample to be processed was prepared in EMEM without FBS. Viral infection was performed with 0.1 mL of the prepared dilution and then the plate should be incubated at 37°C, 5%CO^2^. On the third day of incubation, the media was discarded and replaced with 1 mL of EMEM without FBS. Finally, the plate was incubated under the previously described conditions. The follow-up was daily and the cytopathic effect was recorded. The graphic was created with BioRender.com.

Table 1 shows the comparison between the confluent cell monolayer and the cell suspension infection methods for ZIKV culturing. The suspension method did not require culturing a confluent 2-day monolayer, thus saving time (48 hours) in the process. Interestingly, however, on day 2 after the infection, a complete monolayer of cells was observed in all experiments performed for the suspension-infection method. The suspension method required less medium, less sample volume, and supported multiple samples to be tested (in duplicates and five times more than the monolayer) simultaneously. The suspension method was also 2.7 times less expensive than the monolayer method, which is favorable in resource-limited laboratories (Table 1). Figure 2 shows a visual representation of the culture and inoculation process of the suspension method.

**Table 1:** Comparison of the cost of the cell monolayer versus the cell suspension model for detecting ZIKV.

Characteristics		Monolayer	Suspension
Materials			
	Support	T12.5 culture flask	12-well culture plate
	Medium per sample before the infection (mL)	3	0
	Medium per sample after the infection (mL)	3	2
Cell culture time			
	Prior to infection (hours)	48 ^a^	0
Sample			
	Volume required (mL) ^b^	0.2	0.1
	No. of samples in parallel	1 per flask	5 to 11 per 12-well plate ^c^
Cost per 5 samples (USD) ^d^			
	Total	10.73	4.03

a A monolayer can form within hours if enough cells are planted, thus it is also possible to reduce the time for the formation of a cell monolayer. ^b^ This volume does not include the duplicate. ^c^ Five samples in duplicates or 11 samples with no duplicates, including one cell control. d Prices were reviewed on July 2022. The costs associated with protective equipment and salaries were not considered.

### Development and standardization

Daily search for CPE confirmed ZIKV infectivity, which was comparable in both isolation methods during the standardization process. Supernatants of both methods were harvested on day 4 after infection for MOIs of 0.1 and 0.01, and on day 5 for MOI of 0.001. Regardless of the viral replication, ZIKV replicated in both cell suspension and cell monolayer methods. ZIKV replication was assessed using RT-PCR assay and plaque method.

**Figure 3:**
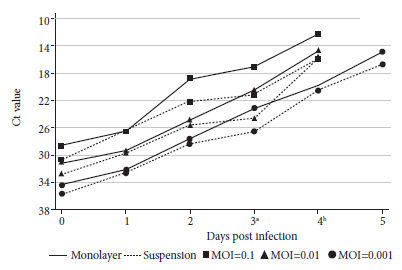
Total viral particles load in confluent and cell suspension methods determined by RT-PCR. The Ct values (y-axis) for confluent cell monolayer (solid line) and cell suspension (dotted line) methods for each sampling day (x-axis) are shown. Kinetics of the 0.1 (square), 0.01 (triangle) and 0.001 (circle) MOI dilutions are also shown. The standard deviation for all experiments ranged from 0.63 to 0.05. Mann-Whitney tests showed no significant difference between methods on days 0, 1, 2 and 5 (p>0.050). a p-value = 0.127, b p-value = 0.275.

Supernatant Ct values were compared daily for each method in order to evaluate the amount of replicated viral particles. We did not find differences in the total number of viral particles between the methods across different MOIs (Figure 3).

**Figure 4:**
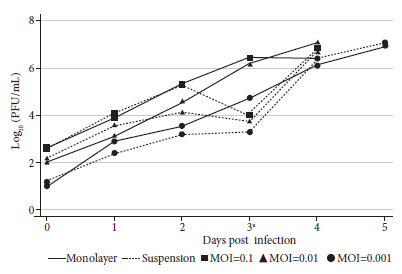
Infectious viral particles load in confluent and cell suspension methods determined by plaque assay. Log10 (PFU/mL) values (y-axis) and their error bars (standard deviation) for confluent cell monolayer (solid line) and cell suspension (dotted line) isolation methods for each day of sampling (x-axis) are shown. Kinetics of the 0.1 (square), 0.01 (triangle) and 0.001 (circle) MOI dilutions are also shown. Mann-Whitney tests showed no significant differences between methods on days 0, 1, 2 and 4 (p>0.050). ^a^ p-value = 0.049.

The analysis of the log_10_ (PFU/mL) values estimated by the plaque assay did not show any differences in the infectious viral particle loads until the second day post-infection (Figure 4). The number of infectious viral particles was lower for the cell suspension method in comparison to the confluent cell monolayer method on day 3 before the media was changed (p=0.049, 3dpi, Figure 4). After the media was changed, the infectious particle load was comparable between the methods across different MOIs (Figure 4).

### Validation of the cell suspension-infection method

We used 29 serum samples from febrile cases to assess the performance of the cell suspension method (Table 2). Overall, we did not observe differences (p=0.243) in the harvest day between the monolayer (9.2 ± 1.3 days) and cell suspension method (9.6 ± 1.1 days), nor between ZIKV-positive (p=0.403) or ZIKV-inconclusive serum samples (p=0.166). The sensitivity in ZIKV-positive samples was 40.0% (6/15, 95%CI: 16.3% - 67.7%) for both methods, but the suspension method showed lower Ct values on the harvest day. Interestingly, two Zika cases were positive by the monolayer method but negative by the cell suspension method, and two other cases were negative by the monolayer but positive by the suspension method. Among the ZIKV-inconclusive samples, the sensitivity was 8.3% (1/12, 95%CI: 0.2% - 38.5%) for the monolayer method and 33.3% (4/12, 95%CI: 9.9% - 65.1%) for the cell suspension method. Both isolation methods were able to detect ZIKV in the two serum samples that were presumptively negative by RT-PCR.

**Table 2:** Validation results of the developed cell suspension-infection method.

Sample code		Ct value in serum/blood	Harvest day		Ct values in Supernatant ^a^	
			Monolayer	Suspension	Monolayer	Monolayer
Positive serum by RT-PCR (n=15) ^b^						
	FHT01120	26.5	7	6	12.6	12.6
	FPI15198	20.9	7	6	15.9	15
	FPI15263	20.3	10	8	22.8	19.2
	FCC00093	28.5	8	8	20.2	16.3
	FPI16318	21.8	10	10	22.1	Negative
	FPY00911	23.9	7	10	26.4	Negative
	FVM00251	25.7	10	10	Negative	30.9
	FHT01144	29.6	7	10	Negative	17.6
	FCC00110	27.1	10	10	Negative	Negative
	FHT01166	24.4	10	10	Negative	Negative
	FPI15173	22.4	10	10	Negative	Negative
	FPI15283	25.1	10	10	Negative	Negative
	FPI15452	23.8	10	10	Negative	Negative
	FHT01166	24.4	10	10	Negative	Negative
	FPI15263	20.3	7	10	Negative	Negative
Inconclusive serum by RT-PCR (n=12) ^c^						
	FPI16332	35.9	10	10	24.3	20.4
	FCC00100	36.1	7	10	Negative	33.1
	FPI16096	38.3	10	10	Negative	19.8
	FPY00920	37.2	10	10	Negative	33.7
	FCC00087	35.2	7	10	Negative	Negative
	FPI15509	37.3	10	10	Negative	Negative
	FPI15698	35.9	10	10	Negative	Negative
	FPI16402	37.1	10	10	Negative	Negative
	FPY00937	36.4	10	10	Negative	Negative
	FPI16290	37.8	10	10	Negative	Negative
	FPY00925	38.1	10	10	Negative	Negative
	FPI15575	31.9	10	10	Negative	Negative
Negative serum by RT-PCR (n=2) ^d^						
	FPI15718	39.8	10	10	29.5	28.1
	FPI16191	39.7	10	10	28.5	27.8

a The real-time reverse transcription-polymerase chain reaction (RT-PCR) was performed in supernatants on harvest day, and cycle threshold (Ct) of duplicates were summarized using means (the standard deviation between duplicates varied between 0.31 and 0.07). ^b^ Ct values ranged from 20.3 to 29.6. ^c^ Ct values for serum/blood samples classified as inconclusive (Ct>34.6) ranged from 35.2 to 38.3. ^d^ Ct values for serum/blood samples classified as negative, since they were very close to the total 40 cycles of amplification.

## DISCUSSION

The isolation method is an important component of ZIKV research. Nonetheless, several researchers have reported failed virus isolation attempts when working with RT-PCR-positive human body fluids due to the time of sample collection after symptom onset and the viral load ^9^^,^^22^^,^^31^. There is a need to improve and develop cost-effective virus culturing methods to provide evidence and duration of infectious virus from body fluids ^18^^,^^20^. Here we report the development and standardization, as well as the preliminary validation of an in-house cell suspension method for ZIKV using Vero cells.

Nikolay *et al*. previously suggested that Vero cells infected with ZIKV at low concentrations and maintained in suspension resulted in the production of large amounts of ZIKV ^26^. This finding led us to utilize the suspension-infection method described in this report. Overall, ZIKV replication was comparable in both culturing methods. Interestingly, two RT-PCR -positive samples (FPI16318 and FPY00911) were only detected by the monolayer method, and two other RT-PCR-positive samples (FVM00251 and FHT01144) were only detected by the suspension method. Despite the discrepancies for these RT-PCR positive samples, it is likely that they do not pose a significant disadvantage for the culture methods since there is usually no need to culture RT-PCR positive samples in real and emergency situations. As described in previous research ^32^, these findings could be explained by differences in the analytical performance of each culturing method, as well as by factors related to the specimens or viral infectivity that were not evaluated here. Though, as expected, the cell suspension method provided several advantages: shorter time, reduced sample volume, and costs per sample. Interestingly, we detected lower infectious viral particle load in the three MOIs tested with the cell suspension method in comparison to the confluent cell monolayer on the third day post-infection, followed by an increase in viral load on the fourth day. This difference was not found when Ct values of both methods were compared, which indicates that RT-PCR assays can detect both non-infectious and infectious viruses while plaque assays detect only infectious particles. Therefore, media changes on the third day most likely favored viral replication as viral load increased on days 4 and 5.

We assessed the performance of the cell suspension method using a set of samples characterized by ZIKV-specific RT-PCR. Clinical samples were obtained from individuals who met the eligibility criteria and had symptoms (such as headache, myalgia) for 5 days or less. We found that there was no difference in the performance of the cell suspension method and the confluent cell monolayer for culturing the virus in the subset of ZIKV-positive samples. Clinical samples with viral loads below RT-PCR’s limit of detection or mutations in the primer- or probe-binding site may incorrectly classify cases as negative or inconclusive ^33^^-^^35^. During the validation process, both isolation methods were able to detect ZIKV in two cases initially classified as negatives by a ZIKV-specific RT-PCR that had clinical symptoms compatible with Zika disease. This finding suggests that the analytical sensitivity of both isolation methods was comparable even in cases with nearly undetectable viral load by virus-specific molecular tools. Restricting the analysis to clinical samples with inconclusive results for detecting ZIKV, the cell suspension method was four times more sensitive in comparison to the monolayer method.

The cell suspension method was preliminary validated using clinical samples collected in four countries where active ZIKV transmission has been reported ^13^^,^^24^. The small number of clinical samples used in this study resulted in wide confidence intervals. Nevertheless, despite the small sample size, we were able to detect a substantial difference in the sensitivity between both culturing methods when the analysis was restricted to ZIKV-inconclusive samples, but comparable with ZIKV-positive and -negative samples. Hence, the use of the cell suspension method may favor the increase of the isolation rate, particularly in inconclusive cases of Zika infection by RT-PCR. The time to form the monolayer can be reduced if enough cells are planted, so no difference in time would exist between both isolation methods. Furthermore, since the suspension method requires changing the media, cross-contamination is possible, particularly in settings with limited resources. Finally, based on the study design, both methods required culturing the virus for up to 10 days, as well as follow-up to confirm identity of the virus. However, molecular detection of the virus could be performed even before 10 days using supernatants.

The epidemiological significance of ZIKV is undeniable and its impact on public health worldwide is an ongoing concern. Therefore, the relevance of developing and validating methodologies to investigate ZIKV in a rapid and efficient manner is of great importance ^14^^-^^16^. Thus, the method described here could serve as an invaluable tool for the detection of ZIKV in existing surveillance systems as well as during outbreaks. Furthermore, the method could contribute to improve ZIKV prevention and control measures by allowing reliable and rapid detection, which would ultimately translate into early identification of cases and timely treatment. Moreover, it is important to highlight that this method has the potential to be used for the detection and isolation of other arboviruses (such as dengue virus, Chikungunya virus) and other pathogens of public health relevance. Similarly, future studies could optimize the preliminary validation described here, as well as expand the proof-of-concept technique using several different cell lines (such as A549, BHK, C6/36, among others) ^18^^,^^25^^,^^26^.

In conclusion, the cell suspension method has several advantages in comparison to the confluent cell monolayer method for detecting ZIKV. Our findings suggest that both culturing methods could be used interchangeably in confirmed cases of Zika disease with detectable viral load by RT-PCR. More importantly, the cell suspension method was four times more sensitive in detecting Zika in inconclusive cases by RT-PCR when compared to a confluent cell monolayer method. The development of this alternative tool, without needing a cell monolayer, could significantly improve the detection capabilities for ZIKV infections in urgent situations.
