# Supplementary material for: Development and validation of a vero cell-based suspension method for the detection of Zika virus
Source: Rev Peru Med Exp Salud Publica. 2023 Sep 25;40(3):297–306. doi: 10.17843/rpmesp.2023.403.12606 (PMC10959515; doi:10.17843/rpmesp.2023.403.12606)
Supplement: Translation

# Translation: Desarrollo y validación de un método de suspensión basado en células Vero para la detección del virus Zika

**Authors:** Dina Popuche, Alfredo Huaman, Steev Loyola, María Silva, Sarah A. Jenkins, Carolina Guevara

**Affiliations:** 1 U.S. Naval Medical Research Unit SOUTH, Lima, Perú; 2 Facultad de Medicina, Universidad Peruana Cayetano Heredia, Lima, Perú; 3 Vysnova Partners Inc., Maryland, EE.UU; 4 Naval Medical Research Center, Silver Spring, EE.UU.

**Keywords:** Zika Virus, Infección por el Virus Zika, Replicación Viral, Técnicas de Cultivo de Célula

## Abstract

**Objetivo:**

. Desarrollar y validar un método de suspensión celular utilizando células Vero 76 para el cultivo del virus Zika (ZIKV) basado en la infección de células recién sembradas no adheridas.

**Material y métodos:**

. Se utilizaron tres multiplicidades de infección diferentes del ZIKV para desarrollar y comparar este novedoso método con el método estándar de monocapa de células confluentes. Además, validamos preliminarmente el método de suspensión utilizando muestras clínicas caracterizadas como positivas o negativas para el ZIKV. El método estándar de monocapa se utilizó como método de referencia, y el aislamiento viral se confirmó mediante un RT-PCR específico del ZIKV. Se estimó la sensibilidad e intervalos de confianza del 95% para el método de suspensión. Asimismo, se realizó una comparación técnica del método de suspensión contra el método de monocapa.

**Resultados:**

. Nuestros hallazgos sugieren que tanto la carga viral como la replicación del ZIKV fueron comparables entre los métodos de infección en monocapa y en suspensión. Aunque ambos métodos fueron adecuados para cultivar y aislar el ZIKV, el método de suspensión se caracterizó por ser más fácil, barato y rápido, así como una técnica de aislamiento sensible. En comparación con el método de monocapa, el método de suspensión fue cuatro veces más sensible en la detección del ZIKV en casos inconclusos por RT-PCR.

**Conclusiones:**

. El método de suspensión tiene el potencial de ser un método eficaz para cultivar y aislar el ZIKV y su uso es potencialmente útil tanto en la investigación como en entornos clínicos.

## INTRODUCCIÓN

El virus Zika (ZIKV) es un flavivirus emergente transmitido por el mosquito *Aedes* en varios países latinoamericanos ^1^^,^^2^. Desde el 2015, múltiples países notificaron la transmisión autóctona del ZIKV, lo cual conllevó a graves brotes en América ^3^^-^^5^. La infección por ZIKV causa manifestaciones clínicas en alrededor del 20% de los pacientes; inicio agudo de fiebre, erupciones maculopapulares, artralgia o conjuntivitis ^3^^,^^6^. Tras la aparición del ZIKV en Brasil en 2015, la infección por el virus se asoció a un aumento significativo del número de bebés nacidos con microcefalia y defectos congénitos, así como a un aumento de enfermedades neurológicas en todos los grupos de edad, incluyendo mielitis y Guillain-Barré ^7^^,^^8^. Diversos estudios han demostrado que el ZIKV puede transmitirse por vía sexual y por transfusión. Esta complejidad en los modos de transmisión plantea un desafío adicional, dado que las personas infectadas pueden no ser conscientes de su capacidad de transmitir el virus ^9^^-^^13^. La declaración del ZIKV como emergencia por parte de la Organización Mundial de la Salud estableció que los países vulnerables a la emergencia del ZIKV deben establecer y mantener la capacidad de detectar y confirmar los casos de ZIKV, independientemente de si se detectan o no casos activos en regiones endémicas ^14^^-^^16^.

Las pruebas diagnósticas de infecciones arbovirales tienen como objetivo detectar el virus o la respuesta inmune basada en los anticuerpos del huésped ^17^. Los ensayos serológicos son utilizados para detectar anticuerpos IgM contra el ZIKV y confirmar una infección reciente; no obstante, los resultados no siempre confirman la infección debido a falsos positivos generados por la reactividad cruzada entre flavivirus ^18^^,^^19^. La detección del ZIKV se ha logrado mediante el uso de la reacción en cadena de la polimerasa con transcripción reversa en tiempo real (RT-PCR) en suero, sangre, orina y otros fluidos corporales; sin embargo, el uso de herramientas de diagnóstico molecular puede ser afectado por la cinética de la viremia y las secuencias que detectan el ARN viral pueden no ser lo bastante específicas para detectar el virus ^20^. Por ello, los resultados negativos o no concluyentes obtenidos en ensayos moleculares no implican necesariamente la ausencia de infección por ZIKV ^16^^,^^18^^,^^21^^,^^22^.

Las técnicas de aislamiento viral mediante diversos cultivos celulares pueden desempeñar un papel importante en el diagnóstico cuando las muestras de casos clínicamente sospechosos son negativas o no concluyentes por RT-PCR. El aislamiento viral es especialmente sensible y útil para detectar virus circulantes durante la fase virémica de la infección y para clasificar correctamente muestras con resultados no concluyentes, pero presenta inconvenientes de uso en entornos de recursos limitados. Aunque el aislamiento del virus puede ser rentable y utilizarse para evaluar la cinética de las infecciones víricas y los resultados clínicos, la técnica requiere operadores altamente calificados y puede ser laboriosa ^15^^,^^17^. El aislamiento del ZIKV puede ser comparativamente complejo ^20^^,^^23^ debido a que este método depende de infraestructura y recursos específicos, procesos de cadena de frío para la preservación viral, laboratorios y suministros de cultivo celular, líneas celulares y personal capacitado y competente, los cuales no necesariamente están inmediatamente disponibles en países en desarrollo. Por lo tanto, la accesibilidad y precisión de los métodos de diagnóstico cobran gran importancia. En específico, existe una necesidad urgente de exámenes diagnósticos para ZIKV que sean confiables, especialmente para las poblaciones susceptibles en riesgo de infección por ZIKV, como las mujeres embarazadas y los bebés ^7^^,^^11^^,^^16^^,^^24^.

El objetivo de este estudio fue encontrar un método de aislamiento que equilibre la necesidad crítica de precisión y la rápida detección del ZIKV, especialmente en situaciones de emergencia o brotes en las que se requiere detección y aislamiento temprano para el desarrollo o evaluación de diferentes pruebas diagnósticas. Por ejemplo, en situaciones de alta sospecha de infección por ZIKV con resultados de RT-PCR negativos o no concluyentes, se requieren métodos complementarios para detectar o aislar rápidamente el virus. En este sentido, los métodos de cultivo convencionales requieren de una monocapa previamente formada, lo que, dependiendo de la concentración de células sembradas, podría llevar entre 12 a 48 horas. Por ello, el desarrollo de métodos de aislamiento que permitan una respuesta casi inmediata sin necesidad de una monocapa previamente formada podría tener un impacto significativo en los tiempos de respuesta y podría incluso impulsar medidas de salud pública de forma temprana. Aquí describimos el desarrollo y la estandarización de un método de suspensión celular, así como su validación preliminar utilizando muestras clínicas humanas. Además, el método de suspensión celular se comparó con el método de monocapa celular confluente utilizando células Vero 76 para optimizar una técnica de aislamiento alternativa, precisa y rápida para la detección del ZIKV.

**MENSAJES CLAVE**

Motivación para realizar el estudio. Búsqueda y desarrollo de nuevas alternativas útiles en la investigación del virus del Zika (ZIKV) en situaciones de emergencia.

Principales hallazgos. Se desarrolló y validó un método novedoso para el aislamiento y cultivo del ZIKV basado en la infección de células Vero recién sembradas no adheridas. Este método se caracterizó por ser fácil, barato, rápido, y más sensible en comparación con el método estándar utilizado para el aislamiento de ZIKV.

Implicancias. El método desarrollado podría ser utilizado para fortalecer el diagnóstico y vigilancia pública, generando así resultados rápidos y confiables.

## MATERIALES Y MÉTODOS

### Diseño de estudio

Estudio experimental basado en el diseño y validación de una prueba de concepto en dos fases; experimentos *in vitro* usando una semilla viral, y validación preliminar usando muestras clínicas. En ambas fases se usaron células Vero (Epitelio renal del mono verde africano [*Chlorocebus*]) obtenidas de la Colección Americana de Cultivos Tipo (ATCC; CRL-1587). Para la validación preliminar se utilizó un total de 29 muestras, las cuales fueron recolectadas en Honduras (códigos FHT, n=4), Colombia (códigos FCC, n=4), Venezuela (código FVM, n=1) y Perú (códigos FPI y FPY, n=20) por investigadores de la Unidad de Investigación Médica Naval SUR de EE.UU. Las muestras fueron obtenidas de individuos con fiebre de hasta cinco días y síntomas clínicos compatibles con la infección por Zika.

### Cultivo celular para la inoculación del ZIKV

Vero 76 es una línea celular óptima para el aislamiento del ZIKV y también sirve para el desarrollo de vacunas víricas humanas y la investigación biomédica de enfermedades virales ^18^^,^^25^^,^^26^. Esta línea celular se utiliza rutinariamente en nuestro laboratorio para el cultivo y aislamiento viral porque es susceptible a los flavivirus como el ZIKV y otros arbovirus. En todos los experimentos celulares se evaluó la viabilidad utilizando azul tripán en un hemocitómetro, obteniéndose valores entre el 90-95%. No se observaron diferencias en la viabilidad celular entre las células utilizadas en el método de monocapa de células confluentes o en el de suspensión celular.

*Método de monocapa celular confluente*

Las células Vero 76 fueron sembradas a una densidad de 1,0 x 10^5^ células/mL en frascos de cultivo T12.5 (Corning, Glendale, AZ, Estados Unidos; Nº de catálogo: 353018) utilizando 3 mL de medio esencial mínimo de Eagle (MEME) (Quality biological, Gaithersburg, MD, Estados Unidos; Nº de catálogo: 112-018-101) suplementado con 10% de suero bovino fetal (SBF) (Merck, Darmstadt, Alemania; Cat. No.: F4135) e incubado durante dos días a 37°C, 5% CO_2_. La monocapa de dos días fue confluente a una densidad de 1,3 x 10^6^ células por frasco. Este método de cultivo estándar se utiliza rutinariamente para preparar células e intentar aislar ZIKV y otros arbovirus.

*Método de suspensión celular*

El día de la inoculación con ZIKV, se sembraron células Vero 76 en placas de cultivo de 12 pozos (Thermo Fisher, Cleveland, OH, Estados Unidos; Nº de cat.: 150628) utilizando 1mL de MEME suplementado con 10% de SBF a una densidad de 2,0 x 10^5^ células/mL. Para facilitar la estabilización del pH, antes de la inoculación, las células fueron incubadas durante 30 minutos a 37°C, 5% CO_2_. Este método se diseñó para eliminar el tiempo de incubación necesario para preparar una monocapa celular confluente y con el fin de tener un tiempo de respuesta más rápido para las pruebas del ZIKV. Sin embargo, es importante mencionar que, a medida que transcurre el tiempo de incubación, las células proliferan y tienden a formar una monocapa. Las placas de cultivo de 12 pozos fueron seleccionadas porque permiten maximizar el uso de células y reactivos por muestra, y porque permiten la inoculación de múltiples muestras por placa.

### Inoculación del virus y seguimiento

La infección viral para ambos métodos se llevó a cabo utilizando una semilla viral de ZIKV aislada en células Vero 76, la cual fue obtenida a partir de una muestra de suero aguda colectada de un caso de Zika en Iquitos, Perú. Para evaluar la replicación viral e inocular el virus en células Vero 76, se prepararon tres multiplicidades de infección (MDI) de 0,1; 0,01; y 0,001 en MEME sin SBF. Los cultivos celulares sin inóculo viral fueron utilizados como controles negativos.

*Método de infección en monocapa*

Después de dos días, el sobrenadante de las células Vero se desechó utilizando una técnica estéril. A continuación, se inocularon las células con 0,2 mL de cada MDI de ZIKV por duplicado. Los frascos se agitaron manual y suavemente de un lado a otro cinco veces y se incubaron durante 1 hora a 37°C en 5% de CO_2_. Después, se añadió 3 mL de MEME suplementado con 2% de SBF a cada cultivo, y se incubaron en las mismas condiciones descritas anteriormente. Para este método, no se realizaron cambios de medio después de la infección con el inóculo viral, por lo que la incubación fue continua.

*Método de infección en suspensión*

La infección se produjo en células que fueron recientemente sembradas y no totalmente adheridas a las placas de cultivo. Este método se estandarizó inoculando 0,1 mL por duplicado de cada una de las tres MDI directamente en cada cultivo de células Vero, los cuales fueron incubados durante tres días a 37°C en 5% de CO_2_. Un estudio previo sugirió que, independientemente del título viral utilizado para la infección, la ausencia de SBF se correlaciona con altas tasas de replicación viral ^27^. Por tanto, para evitar el agotamiento de los componentes del medio, al tercer día se retiró cuidadosamente el medio con una pipeta de 1 mL sin alterar la monocapa, y se sustituyó con 1 mL de MEME nuevo sin SBF. Luego, los cultivos se colocaron en la incubadora a 37°C con un 5% de CO_2_.

*Seguimiento*

Los cultivos de células Vero inoculados con ZIKV fueron observados diariamente para detectar efectos citopáticos (ECP), y se colectaron 200 µL de sobrenadante de cada cultivo inoculado para su posterior análisis molecular y por el ensayo de placa viral. Los cultivos se cosecharon cuando las células mostraron >75% (3+) de ECP o hasta 10 días después de la inoculación.

### Ensayo molecular

Se extrajo ARN viral utilizando 140 µL de cada sobrenadante recolectado diariamente de células Vero infectadas con ZIKV a partir del día 0 hasta el momento del desarrollo de ECP o hasta 10 días después de la inoculación. La extracción se realizó utilizando el kit QIAamp Viral RNA (Qiagen, Hilden, Alemania, Cat. No.: 52904) de acuerdo con las instrucciones del fabricante. La detección del ARN del ZIKV se realizó utilizando cebadores y sondas, así como condiciones de ciclado previamente descritos ^28^. La mezcla de reacción para la RT-PCR de un paso se preparó utilizando Fast Virus 1-step (Thermo Fisher, Cleveland, OH, Estados Unidos; Nº de cat.: 4444436), y la amplificación se realizó en el instrumento Applied Biosystems 7500 Fast Real-Time PCR (Thermo Fisher, Cleveland, OH, Estados Unidos; Nº de cat.: 4406984). El límite de detección del ensayo RT-PCR para ZIKV ha sido previamente descrito en el umbral de ciclo (Ct) de 36,2 ± 1,6 ^28^. En este estudio, las muestras de suero o sangre con un valor Ct inferior o igual al valor de corte fueron clasificadas como positivas (Ct ≤ 34,6), mientras que aquellas sin señal de amplificación fueron clasificadas como negativas. Las muestras con una señal de amplificación tardía (Ct > 34,6) fueron clasificadas como no concluyentes. Los valores de Ct fueron registrados y utilizados como referencia de la carga viral ***.***

### Ensayo de placa viral

Las células A549 y Vero son susceptibles a la infección por ZIKV, dando lugar al ECP ^25^. En experimentos previos realizados en nuestro laboratorio, no hemos observado diferencias significativas en la carga de ZIKV al comparar los recuentos de unidades formadoras de placas en células Vero y A549 utilizando el método semisólido de ensayo de placa viral ^29^. Sin embargo, observamos que la mayoría de las placas se formaban mejor en las células A549 al tercer día de la infección en comparación con las células Vero (Figura 1). El método semisólido fue ejecutado preparando una suspensión de células A549 en placas de 12 pozos a una densidad de 2,0 x 10^5^ células/mL por pozo. Los cultivos celulares se colocaron en la incubadora durante 30 minutos a 37°C y 5% de CO_2_. Las muestras de sobrenadante celular se analizaron en placas de cultivo de 12 pozos, tal como ha sido previamente descrito ^30^. Seis diluciones 1/10 fueron preparadas utilizando 100 µL de cada muestra de sobrenadante, y luego se inocularon en los cultivos por duplicado. Después de tres horas de incubación en las mismas condiciones descritas anteriormente, se añadió 1 mL de carboximetilcelulosa al 3% a cada cultivo, y luego, se incubó durante tres días. Las células se tiñeron utilizando 3 mL de una solución compuesta por acetato de sodio anhidro, negro azul de naftol y ácido acético. Las unidades formadoras de placa (UFP) fueron contabilizadas, y se utilizaron para estimar las UFP por mL (UFP/mL) y determinar el título viral infeccioso.

**Figura 1:** Ensayo de placa usando virus del Zika (ZIKV) en células Vero 76 y A549. Se sembró ZIKV en células Vero 76 y A549 para determinar la línea celular óptima para el recuento. En todos los paneles se presentan monocapas celulares teñidas en días diferentes. Las placas de ZIKV en células Vero 76 (A y B) y A549 (C y D) se tiñeron después de 5 y 3 días posteriores a la infección, respectivamente. Las flechas rojas representan placas bien formadas consideradas para el recuento. Las flechas verdes representan placas pequeñas y no bien formadas.

### Validación del método de infección en suspensión celular

Se utilizaron 29 muestras de sueros agudos para validar el método de suspensión celular. Este conjunto de especímenes incluyó 15 muestras positivas para ZIKV, 12 muestras inconclusas para ZIKV y 2 muestras presuntamente negativas para ZIKV según lo determinado por RT-PCR ^28^. Las muestras inconclusas se incluyeron en la validación bajo el supuesto de que la amplificación tardía (39,0 > Ct > 34,6) observada en la RT-PCR estaba asociada a una carga viral baja y que la propagación en cultivo celular podría ayudar posteriormente a clasificar la muestra como positiva o negativa. Las dos muestras negativas se incluyeron porque mostraban un pequeño patrón de amplificación cercano a los 40 ciclos. Las muestras de sangre se recogieron de participantes durante la fase aguda de la enfermedad febril, los cuales manifestaron síntomas clínicos compatibles con la infección por Zika. Las muestras recolectadas fueron centrifugadas para separar el suero y se almacenaron a -80°C hasta su uso. Las muestras de suero descongeladas se diluyeron 1/10 utilizando MEME sin SBF, y la infección se llevó a cabo en duplicados en los métodos de infección por suspensión celular e infección por monocapa confluente, tal y como se describe líneas arriba.

### Análisis estadístico

Los valores de las UFP/mL se transformaron a log_10_ (UFP/mL). Los valores de Ct y log_10_ (UFP/mL) fueron utilizados de forma independiente para evaluar la cinética de la replicación viral en el método de monocapa de células confluentes y de suspensión celular con las tres MDI. Se realizaron pruebas de Mann-Whitney utilizando los valores Ct y log_10_ (UFP/mL) por día sin tener en cuenta el inóculo de cada MDI para explorar diferencias entre los métodos. En la validación, exploramos las diferencias en el día de cosecha utilizando una prueba T pareada. La sensibilidad e intervalos de confianza al 95% (IC95%) para ambos modelos de infección fueron estimados considerando el ensayo molecular como la referencia. El análisis de los datos se realizó utilizando Stata v16.0 (StataCorp. 2015. Stata Statistical Software: Release 14. College Station, TX: StataCorp LP.; licenciado por la Universidad Peruana Cayetano Heredia) y considerando p<0,05 como significativo.

### Aspectos éticos

El estudio se llevó a cabo de acuerdo con la Declaración de Helsinki. Los protocolos de estudio NMRCD.2010.0010 y NAMRU6.2020.004 fueron aprobados por la Junta de Revisión Institucional de la Unidad de Investigación Médica Naval SUR de EE.UU. en cumplimiento de todas las normativas federales y locales aplicables que rigen la protección de los seres humanos. El primer protocolo permitió la recolección de muestras de individuos que emitieron consentimiento informado para la investigación de arbovirus, mientras que el segundo protocolo permitió la utilización de muestras anonimizadas para el desarrollo y validación de pruebas desarrolladas en el laboratorio.

## RESULTADOS

### Análisis técnico

En la Tabla 1 se comparan los métodos de cultivo del ZIKV en monocapa de células confluentes y de células en suspensión. En específico, el método de suspensión no requirió el cultivo de una monocapa confluente de 2 días; ahorrando así tiempo (48 horas) en el proceso. Interesantemente, al día 2 después de la infección se observó una monocapa celular completa en todos los experimentos realizados para el método de infección por suspensión. El método de suspensión requirió menos medio, menos volumen de muestra y permitió analizar múltiples muestras (cinco veces más, y por duplicados en comparación al de monocapa) simultáneamente. El método de suspensión también fue 2,7 veces menos costoso que el método de monocapa, lo que resulta favorable en laboratorios con recursos limitados (Tabla 1). En la Figura 2, se muestra una representación gráfica del proceso de cultivo e inoculación del método de suspensión.

**Tabla 1:** Comparación técnica del método de monocapa celular frente al de suspensión celular para la detección del ZIKV

Características		Monocapa	Suspensión
Materiales			
	Soporte	Frasco de cultivo T12.5	Placa de cultivo de 12 pozos
	Medio por muestra antes de la infección (mL)	3	0
	Medio por muestra tras la infección (mL)	3	2
Tiempo de cultivo celular			
	Antes de la infección (horas)	48 ^a^	0
Muestra			
	Volumen necesario (mL) ^b^	0,2	0,1
	N° de muestras en paralelo	1 por frasco	5 a 11 por placa de 12 pozos ^c^
Costo por 5 muestras (USD) ^d^			
	Total	10,73	4,03

a La monocapa puede formarse en cuestión de horas si se siembran suficientes células, por lo que es posible reducir el tiempo de formación de la monocapa. ^b^ El volumen indicado no incluye el duplicado. Si se realiza un duplicado del método de la monocapa, se requiere un frasco de cultivo adicional, mientras que las placas de 12 pozos podrían permitir hasta 5 muestras por duplicado. ^c^ Cinco muestras por duplicado u 11 muestras sin duplicados, incluido un control de células. ^d^ Los precios se revisaron en julio de 2022. No se tuvieron en cuenta los costos asociados a los equipos de protección y salarios.

**Figura 2:** Flujo general del proceso de cultivo e inoculación viral del método de suspensión celular. El día de la inoculación viral, las células Vero 76 deben sembrarse en una placa de cultivo de 12 pozos a una densidad de 2,0 x 105 células/mL en medio esencial mínimo de Eagle (MEME) suplementado con 10% de suero bovino fetal (SBF). La placa debe incubarse durante 30 minutos a 37°C, 5% CO2. La muestra a procesar debe ser diluida 1:10 en MEME sin SBF. La infección viral debe realizarse con 0,1 mL de la dilución preparada y luego la placa debe incubarse a 37°C, 5% CO2. Al tercer día de incubación, se desecha el medio y se sustituye por 1 mL de MEME sin SBF. Por último, la placa debe incubarse en las condiciones descritas anteriormente. El seguimiento debe ser diario y se debe registrar el efecto citopático. La ilustración fue creada con BioRender.com.

### Desarrollo y estandarización

La infecciosidad del ZIKV se demostró mediante observaciones diarias del ECP, la cual fue comparable en ambos métodos de aislamiento durante el proceso de estandarización. Los sobrenadantes de ambos métodos se cosecharon al día 4 después de la infección para la MDI de 0,1 y 0,01, y al día 5 para la MDI de 0,001. Independientemente de la cinética de replicación viral, el ZIKV se replicó tanto en el método de suspensión celular como en el de monocapa celular. La replicación del ZIKV se evaluó mediante RT-PCR y el ensayo de placa viral.

Los valores de Ct en el sobrenadante se compararon diariamente para cada método con el fin de evaluar la cantidad de partículas virales producidas. En general, no se detectaron diferencias en el número total de partículas virales entre los métodos y las diferentes MDI (Figura 3).

**Figura 3:** Carga total de partículas virales en los métodos de confluencia y suspensión celular determinada por RT-PCR. Se representan los valores Ct (eje y) para los métodos de monocapa celular confluente (línea continua) y suspensión celular (línea discontinua) para cada día de muestreo (eje x). También se presenta la cinética de las diluciones multiplicidades de infección (DMI) de 0,1 (cuadrado), 0,01 (triángulo) y 0,001 (círculo). La desviación estándar de todos los experimentos osciló entre 0,63 y 0,05. La prueba de Mann-Whitney no evidenció diferencias significativas entre los métodos para los días 0, 1, 2 y 5 (p>0,050). a Valor de p = 0,127; b valor de p = 0,275.

El análisis de los valores log_10_ (UFP/mL) estimados por el ensayo de placas virales no mostró diferencias para las cargas de partículas virales infecciosas hasta el tercer día posterior a la infección (Figura 4). El número de partículas virales infecciosas fue inferior para el método de suspensión celular en comparación al método de monocapa celular confluente al día 3, antes de que se cambiara el medio (p=0,049, 3dpi, Figura 4). Después de cambiar el medio, la carga de partículas infecciosas fue comparable entre los métodos en todas las MDI (Figura 4).

**Figura 4:** Carga de partículas virales infecciosas en el método confluente y de suspensión celular determinada por ensayo de placa viral. Se trazaron los valores Log10 (UFP/mL) (eje y) y sus barras de error (desviación estándar) para los métodos de aislamiento en monocapa celular confluente (línea continua) y suspensión celular (línea discontinua) para cada día de muestreo (eje x). También se representó la cinética de las diluciones para cada multiplicidad de infección de 0,1 (cuadrado), 0,01 (triángulo) y 0,001 (círculo). La prueba de Mann-Whitney no evidenció diferencias significativas entre los métodos en los días 0, 1, 2 y 4 (p>0,050). a Valor de p = 0,049.

### Validación del método de infección con células en suspensión

Se utilizó un total de 29 muestras de suero de casos febriles para evaluar el rendimiento del método de suspensión celular (Tabla 2). En general, no se observaron diferencias (p=0,243) en el día de cosecha entre el método de monocapa (9,2 ± 1,3 días) y el de suspensión celular (9,6 ± 1,1 días), ni entre las muestras de suero positivas (p=0,403) o no concluyentes para ZIKV (p=0,166). La sensibilidad en muestras positivas a ZIKV fue del 40,0% (6/15, IC95%: 16,3% - 67,7%) para ambos métodos, pero el método de suspensión dio lugar a valores Ct más bajos en el día de la cosecha. Interesantemente, dos casos de Zika fueron positivos con el método de monocapa pero negativos con el método de suspensión celular, y otros dos casos fueron negativos con el método de monocapa pero positivos con el método de suspensión. Entre las muestras no concluyentes para ZIKV, la sensibilidad fue del 8,3% (1/12, IC95%: 0,2% - 38,5%) para el método de monocapa y del 33,3% (4/12, IC95%: 9,9% - 65,1%) para el método de suspensión celular. Ambos métodos de aislamiento fueron capaces de detectar el ZIKV en las dos muestras de suero presuntamente negativas por RT-PCR.

**Tabla 2:** Resultados de la validación del método de infección en suspensión de células

Código de muestra		Valor de Ct en suero/sangre	Día de cosecha		Valores de Ct en el sobrenadante ^a^	
			Monocapa	Suspensión	Monocapa	Suspensión
Suero positivo por RT-PCR (n=15) ^b^						
	FHT01120	26,5	7	6	12,6	12,6
	FPI15198	20,9	7	6	15,9	15
	FPI15263	20,3	10	8	22,8	19,2
	FCC00093	28,5	8	8	20,2	16,3
	FPI16318	21,8	10	10	22,1	Negativo
	FPY00911	23,9	7	10	26,4	Negativo
	FVM00251	25,7	10	10	Negativo	30,9
	FHT01144	29,6	7	10	Negativo	17,6
	FCC00110	27,1	10	10	Negativo	Negativo
	FHT01166	24,4	10	10	Negativo	Negativo
	FPI15173	22,4	10	10	Negativo	Negativo
	FPI15283	25,1	10	10	Negativo	Negativo
	FPI15452	23,8	10	10	Negativo	Negativo
	FHT01166	24,4	10	10	Negativo	Negativo
	FPI15263	20,3	7	10	Negativo	Negativo
Suero no concluyente por RT-PCR (n=12) ^c^						
	FPI16332	35,9	10	10	24,3	20,4
	FCC00100	36,1	7	10	Negativo	33,1
	FPI16096	38,3	10	10	Negativo	19,8
	FPY00920	37,2	10	10	Negativo	33,7
	FCC00087	35,2	7	10	Negativo	Negativo
	FPI15509	37,3	10	10	Negativo	Negativo
	FPI15698	35,9	10	10	Negativo	Negativo
	FPI16402	37,1	10	10	Negativo	Negativo
	FPY00937	36,4	10	10	Negativo	Negativo
	FPI16290	37,8	10	10	Negativo	Negativo
	FPY00925	38,1	10	10	Negativo	Negativo
	FPI15575	31,9	10	10	Negativo	Negativo
Suero negativo por RT-PCR (n=2) ^d^						
	FPI15718	39,8	10	10	29,5	28,1
	FPI16191	39,7	10	10	28,5	27,8

a La reacción en cadena de la polimerasa con transcripción reversa en tiempo real (RT-PCR) se realizó en sobrenadantes el día de la recolección, y el umbral de ciclo (Ct) de los duplicados se resumió utilizando medias (la desviación estándar entre duplicados varió entre 0,31 y 0,07). ^b^ Los valores de Ct oscilaron entre 20,3 y 29,6. ^c^ Los valores de Ct de las muestras de suero/sangre clasificadas como no concluyentes (Ct>34,6) oscilaron entre 35,2 y 38,3. ^d^ Las muestras de suero/sangre fueron clasificadas como negativas por valores de Ct muy cercanos a los 40 ciclos totales de amplificación.

## DISCUSIÓN

El método de aislamiento es una parte importante de la investigación del ZIKV. No obstante, varios investigadores han informado de intentos fallidos para aislar el virus al trabajar con fluidos corporales humanos positivos por RT-PCR debido al tiempo de recolección de la muestra tras la aparición de los síntomas y a la carga viral ^9^^,^^22^^,^^31^. Existe la necesidad de mejorar y desarrollar métodos de cultivo viral rentables que proporcionen evidencia y permitan evaluar la cinética de virus infecciosos en fluidos corporales ^18^^,^^20^. Aquí informamos del desarrollo y estandarización, así como de la validación preliminar de un método de suspensión celular para ZIKV utilizando células Vero.

Nikolay *et al*. sugirieron previamente que las células Vero infectadas con ZIKV a bajas concentraciones y mantenidas en suspensión daban lugar a la producción de grandes cantidades de ZIKV ^26^. Este hallazgo nos llevó a utilizar el método de infección en suspensión descrito en este documento. En general, la replicación del ZIKV fue comparable en ambos métodos de cultivo. Interesantemente, dos muestras positivas por RT-PCR (FPI16318 y FPY00911) sólo fueron detectadas por el método de monocapa, y otras dos muestras también positivas por RT-PCR (FVM00251 y FHT01144) sólo se detectaron con el método de suspensión. A pesar de las discrepancias observadas para estas muestras positivas por RT-PCR, es probable que el hallazgo no suponga una desventaja significativa para los métodos de cultivo evaluados, dado que, en condiciones reales y de emergencia, normalmente no hay necesidad de cultivar muestras positivas por RT-PCR. Tal como fue previamente descrito ^32^, estos resultados podrían explicarse por diferencias en el rendimiento analítico de cada método de cultivo, así como por factores relacionados a las muestras o la infectividad viral; los cuales no fueron evaluados aquí. Aunque, como era de esperar, el método de suspensión celular ofrece múltiples ventajas: menor tiempo, menor volumen de muestra y menor costo por muestra. Interesantemente, detectamos una menor carga de partículas virales infecciosas en las tres MDI probadas en el método de suspensión celular en comparación con la monocapa celular confluente al tercer día posterior a la infección, seguido de un aumento de la carga viral al cuarto día. Esta diferencia no fue observada al comparar los valores Ct de ambos métodos, lo cual confirma que el RT-PCR puede detectar tanto virus no infeccioso como infeccioso, mientras que el ensayo de placa viral sólo detecta partículas infecciosas. Por tanto, lo más probable es que los cambios de medio al tercer día favorezcan la replicación viral, puesto que la carga viral aumentó al día 4 y 5.

El rendimiento del método de suspensión celular fue evaluado utilizando un conjunto de muestras caracterizadas por un RT-PCR específico para detectar ZIKV. Las muestras clínicas se obtuvieron de individuos que cumplían los criterios de elegibilidad y presentaban síntomas (como fiebre, dolor de cabeza, mialgia) durante 5 días o menos. Encontramos que, en el subconjunto de muestras positivas para ZIKV, no hubo diferencia en el rendimiento del método de suspensión celular y el monocapa de células confluentes para cultivar el virus. Las muestras clínicas con cargas virales por debajo del límite de detección del RT-PCR o mutaciones en el sitio de unión del cebador o la sonda pueden resultar en la clasificación incorrecta de casos negativos o no concluyentes ^33^^-^^35^. En la validación, ambos métodos de aislamiento pudieron detectar ZIKV en dos casos clasificados inicialmente como negativos por el RT-PCR específico para ZIKV, los cuales presentaban síntomas clínicos compatibles con la enfermedad del Zika. Este hallazgo sugiere que la sensibilidad analítica de ambos métodos de aislamiento fue comparable incluso en casos con carga viral casi indetectable por herramientas moleculares que específicamente detectan el virus. Restringiendo el análisis a las muestras clínicas con resultados no concluyentes para la detección del ZIKV, el método de suspensión celular fue cuatro veces más sensible en comparación al método de monocapa.

El método de suspensión celular se validó de forma preliminar utilizando muestras clínicas recolectadas en cuatro países en los que se había notificado transmisión activa del ZIKV ^13^^,^^24^. El reducido número de muestras clínicas utilizadas en este estudio dio lugar a amplios intervalos de confianza. No obstante, a pesar del pequeño tamaño de la muestra, pudimos detectar una diferencia sustancial en la sensibilidad entre ambos métodos de cultivo cuando el análisis se restringió a muestras no concluyentes para ZIKV, pero comparable con muestras positivas y negativas para ZIKV. Por lo tanto, el uso del método de suspensión celular puede favorecer el aumento de la tasa de aislamiento, específicamente en casos no concluyentes de infección por Zika mediante RT-PCR. Por otro lado, el tiempo para formar la monocapa puede reducirse si se siembran suficientes células, por lo que podría no existir diferencia de tiempo entre ambos métodos de aislamiento. Además, dado que el método de suspensión requiere cambiar los medios, es posible que se produzca contaminación cruzada, especialmente en entornos con pocos recursos. Por último, según el diseño del estudio, ambos métodos requirieron del cultivo del virus durante un máximo de 10 días y realizar estudios de seguimiento para confirmar la presencia del virus. Sin embargo, la detección molecular del virus podría realizarse incluso antes de los 10 días utilizando sobrenadantes.

La importancia epidemiológica del ZIKV es innegable y su impacto en la salud pública mundial es una preocupación constante. Por lo tanto, la relevancia de desarrollar y validar metodologías para investigar el ZIKV de manera rápida y eficiente es de gran importancia ^14^^-^^16^^).^ Así, el método descrito aquí podría servir como una herramienta invaluable para la detección de ZIKV en los sistemas de vigilancia existentes, así como en la respuesta a brotes. Además, el método podría contribuir a diversos esfuerzos de prevención y control del ZIKV al proporcionar detecciones fiables y rápidas, que en última instancia se traducen en la identificación temprana de casos y de tratamiento oportuno. Además, es importante destacar que el método descrito tiene potencial de ser utilizado en la detección y aislamiento de otros arbovirus (como el virus del dengue, el virus Chikungunya) y otros patógenos de relevancia para la salud pública. Del mismo modo, futuros estudios podrían optimizar la validación preliminar aquí descrita, así como ampliar la prueba de concepto utilizando líneas celulares diferentes (como A549, BHK, C6/36, entre otras) ^18^^,^^25^^,^^26^.

En conclusión, el método de suspensión celular tiene múltiples ventajas en comparación con el método de monocapa celular confluente para detectar el ZIKV. Nuestros hallazgos sugieren que ambos métodos de cultivo podrían utilizarse indistintamente en casos confirmados de enfermedad por Zika con carga viral detectable mediante RT-PCR. Más importante aún, en comparación con el método de monocapa de células confluentes, el método de suspensión celular fue cuatro veces más sensible en la detección del Zika en casos no concluyentes por RT-PCR. El desarrollo de esta herramienta alternativa, que no requiere de una monocapa celular, mejorará significativamente las capacidades de detección de infecciones por ZIKV en situaciones de emergencia.
